# Assessing the readiness of digital data infrastructure for opioid use disorder research

**DOI:** 10.1186/s13722-020-00198-3

**Published:** 2020-07-10

**Authors:** Arjun Venkatesh, Caitlin Malicki, Kathryn Hawk, Gail D’Onofrio, Jeremiah Kinsman, Andrew Taylor

**Affiliations:** 1 Department of Emergency Medicine, Yale University School of Medicine, 464 Congress Ave, Suite 260, New Haven, CT 06519 USA; 2 Center for Outcomes Research and Evaluation, Yale New Haven Hospital, New Haven, CT USA

**Keywords:** Common data elements, Electronic health records, Opioid-related disorders, Emergency medicine

## Abstract

**Background:**

Gaps in electronic health record (EHR) data collection and the paucity of standardized clinical data elements (CDEs) captured from electronic and digital data sources have impeded research efforts aimed at understanding the epidemiology and quality of care for opioid use disorder (OUD). We identified existing CDEs and evaluated their validity and usability, which is required prior to infrastructure implementation within EHRs.

**Methods:**

We conducted (a) a systematic literature review of publications in Medline, Embase and the Web of Science using a combination of at least one term related to OUD and EHR and (b) an environmental scan of publicly available data systems and dictionaries used in national informatics and quality measurement of policy initiatives. Opioid-related data elements identified within the environmental scan were compared with related data elements contained within nine common health data code systems and each element was graded for alignment with match results categorized as “exact”, “partial”, or “none.”

**Results:**

The literature review identified 5186 articles for title search, of which 75 abstracts were included for review and 38 articles were selected for full-text review. Full-text articles yielded 237 CDEs, only 12 (5.06%) of which were opioid-specific. The environmental scan identified 379 potential data elements and value sets across 9 data systems and libraries, among which only 84 (22%) were opioid-specific. We found substantial variability in the types of clinical data elements with limited overlap and no single data system included CDEs across all major data element types such as substance use disorder, OUD, medication and mental health. Relative to common health data code systems, few data elements had an exact match (< 1%), while 61% had a partial match and 38% had no matches.

**Conclusions:**

Despite the increasing ubiquity of EHR data standards and national attention placed on the opioid epidemic, we found substantial fragmentation in the design and construction of OUD related CDEs and little OUD specific CDEs in existing data dictionaries, systems and literature. Given the significant gaps in data collection and reporting, future work should leverage existing structured data elements to create standard workflow processes to improve OUD data capture in EHR systems.

## Background

The opioid epidemic, which is responsible for nearly 400,000 overdose deaths since 1999, has received increased attention from researchers and policymakers as a leading cause of injury-related death in the United States [1]. Unfortunately, few evidence-based solutions to the epidemic exist due to limited prior attention and investments in research infrastructure for a condition often stigmatized or marginalized [2]. The passage of the Substance Use-Disorder Prevention that Promotes Opioid Recovery and Treatment (SUPPORT) for Patients and Communities Act, however, has generated marked enthusiasm and support to address gaps in research, surveillance, and care for opioid use disorder (OUD) using increasingly available electronic and digital data sources such as electronic health records (EHRs) [3]. While the National Institute of Health encourages the use of common data elements (CDEs) “to improve data quality and opportunities for comparison and combination of data from multiple studies and with electronic health records” [4], numerous challenges still exist in identifying and incorporating OUD-specific CDEs into research initiatives [5, 6]. Prior work has identified numerous gaps in EHRs or data standards that preclude high-quality OUD research, including single site-specific definitions that cannot be generalized for observational studies or surveillance as well as the use of disparate data EHR data systems between vendors when capturing and storing health data [7, 8]. Additionally, fragmented CDEs that are not easily translated across settings or data systems that are inherently designed for select types or structured or clinically oriented data prevent the effective development of quality measurement or surveillance systems [9, 10]. For example a common National Institutes of Health (NIH) CDE is derived from the Timeline Followback Method Assessment, which collects information about opioid use in the past week [11]. However, this CDE does not map to any existing data standard or system which are inherently designed for more structured data or hierarchies of data terms in ontologies not specific to a single question.

The creation and inclusion of opioid relevant CDEs in clinical data registries and EHRs would both enable and improve the quality of substance use disorder research and the evaluation of interventions to improve outcomes [6]. For example, improving EHR data infrastructure for OUD data elements could provide the building blocks for future quality measures, performance benchmarking, and answering important research questions, such as “how many providers provide naloxone or administer buprenorphine for OUD?” or “what proportion of emergency department (ED) patients with OUD have low back pain?” [12], which would improve our understanding of the scope of this issue, as well as evaluate interventions. We therefore aimed to identify and categorize existing CDEs in relation to OUD and assess their alignment with common data standards, which is required prior to infrastructure implementation.

## Methods

This study included the parallel conduct of an environmental scan and a literature review. The former was designed to capture data elements and concepts used in national informatics and quality measurement initiatives, while the latter encompassed CDEs published in peer-reviewed literature. This comprehensive study design was based on the current structure and availability of relevant data, with input from a multidisciplinary committee of experts, and allowed for inclusion of a diverse set of data standards ranging from diagnostic codes originally intended for billing purposes to EHR standards for clinical information. The Yale University Institutional Review Board (IRB) determined that review and approval were not required, as the project did not involve human subjects research.

### Environmental scan

We conducted an environmental scan of publicly available data systems, data elements and data dictionaries used in several public and private initiatives to identify OUD data elements suitable for capture in the EHR. The environmental scan was conducted in concert with guidance of the Centers for Medicare and Medicaid Service’s MMS Blueprint, a guidance document for quality measure development in which environmental scans are similarly applied to diverse data types for similar purposes to this work [13].

### Data sources

We searched publicly available data system and dictionary websites for opioid-related data sets and elements including the Value Set Authority Center (VSAC) [14], Centers for Medicare and Medicaid (CMS) Data Element Library (DEL) [15], National Quality Measures Clearinghouse (NQMC) [16], the NIH CDEs [4], the University of Washington Alcohol and Drug Abuse Institute (ADAI) Library Instruments [17], the National Human Genome Research Institute (NHGRI): PhenX Toolkit [18] and the National Institute of Drug Abuse (NIDA) CDEs [11]. Each source contains fairly unique CDE information including: clinical concepts within the VSAC, quality measure specific data instruments within the DEL, primarily patient reported outcome survey instruments within the NIH CDE, human readable data element specifications within the NQMC, and consensus measurement protocols within PhenX.

### Search strategy

For VSAC, CMS DEL, NQMC, NIH CDEs, University of Washington ADAI, and PhenX researchers searched “opioid,” along with relevant keywords such as *heroin, buprenorphine, naloxone, Narcan* and *methadone*. We found that expanding search terms beyond *opioid* did not return any additional value sets that were not found using *opioid* only. Given the relevance of NIDA CDEs to substance use disorder [11], we manually reviewed all 204 CDEs for any referencing opioids.

### Inclusion/exclusion

For the analysis, we included all data elements considered relevant to OUD research based on a review by two research investigators (CM, AT) and any disagreements in relevance were reviewed by a third investigator (AKV) and resolved by consensus discussion. In general, due to limited specificity of CDEs, the process was inclusive of most data elements and only data elements solely specific to another substance use disorder such as tobacco or alcohol without any OUD relevance were excluded.

### Analysis

Opioid-related data elements identified for each data system and library were compared with related data elements contained within the following common health data code systems: Current Procedural Terminology (CPT), International Classification of Diseases, 9th Revision (ICD9), International Classification of Diseases, 10th Revision (ICD10), Systematized Nomenclature of Medicine Clinical Terms (SNOMEDCT), Logical Observation Identifiers Names and Codes (LOINC), National Drug File –Reference Terminology (NDFRT), Healthcare Common Procedure Coding System (HCPCS), Centers for Disease Control and Prevention Race and Ethnicity Code Set (CDCREC) and RXNORM. Data elements were graded by a study investigator, with match results categorized as “exact”, “partial”, or “none.” To ensure accuracy, matching was reviewed by a second study investigator and any disagreements were resolved by a third investigator.

### Literature review

For the literature review, we constructed a comprehensive search strategy built upon clinical experience, prior systematic reviews in substance use disorder literature, and input from a professional librarian.

### Sources

We conducted a search of relevant publications in Medline and Embase using OVID, as well as the Web of Science.

### Search strategy

Searches included a combination of at least one term related to opioid use disorders and electronic medical records. Opioid related search terms included analgesics, opioid-related disorders, opiate alkaloids, or types of opioids (opioid OR opiate OR heroin OR naloxone OR narcan OR evzio OR percocet OR endocet OR primlev OR oxycontin OR oxycodone OR roxicodone OR xtampza OR oxaydo OR buprenorphine OR buprenex OR butrans OR probuphine OR suboxone OR belbuca). Electronic medical record search terms included medical records, EHR OR Electronic Health Record* OR Electronic Medical Record* OR Electronic Data Element* OR Electronic Phenotype* OR value set authority center* OR VSAC OR ontology OR SNOMED OR ICD9 OR ICD10 OR data standard* OR HL7 OR Health Level 7 OR FHIR OR common data element* OR medical record* OR clinical data element*. Search terms were similar for Web of Science and adapted for their terms/indexes.

### Inclusion/exclusion

**Fig. 1:**
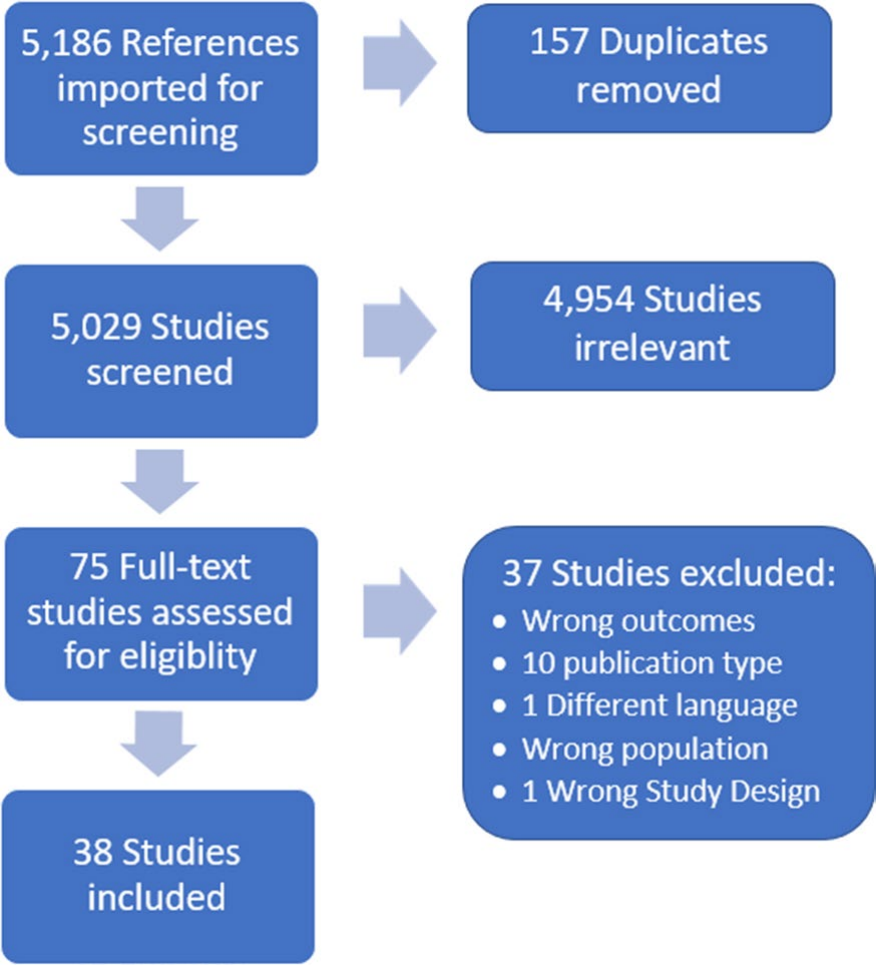
PRISMA flow diagram of literature review

Using Covidence, we systematically reviewed our search results to identify publications for review and analysis, the results of which are presented in a PRISMA flow chart in Fig. 1. In summary, the search returned 5186 references (1070 in Medline, 3653 in Embase and 1103 in Web of Science), and after removing duplicates (n = 157) and articles that did not include relevant content related to both opioid use disorders and electronic medical records (n = 4954), a total of 75 full text articles remained for further analysis. Of these, 37 studies were excluded primarily due to lack of relevant outcomes or non-peer-reviewed publication type and a total of 38 studies were included for review and analysis.

### Analysis

We conducted a systematic assessment of included studies based on expert review, abstraction and curation by two study investigators. Each CDE identified in the manuscript was abstracted into a standardized data collection tool and classified each data element as related to diagnosis, medication, patient demographics, or vital signs. Given the heterogeneity of underlying studies as well as the purpose of this exploratory literature review, no meta-analysis was considered necessary or feasible.

## Results

**Fig. 2:**
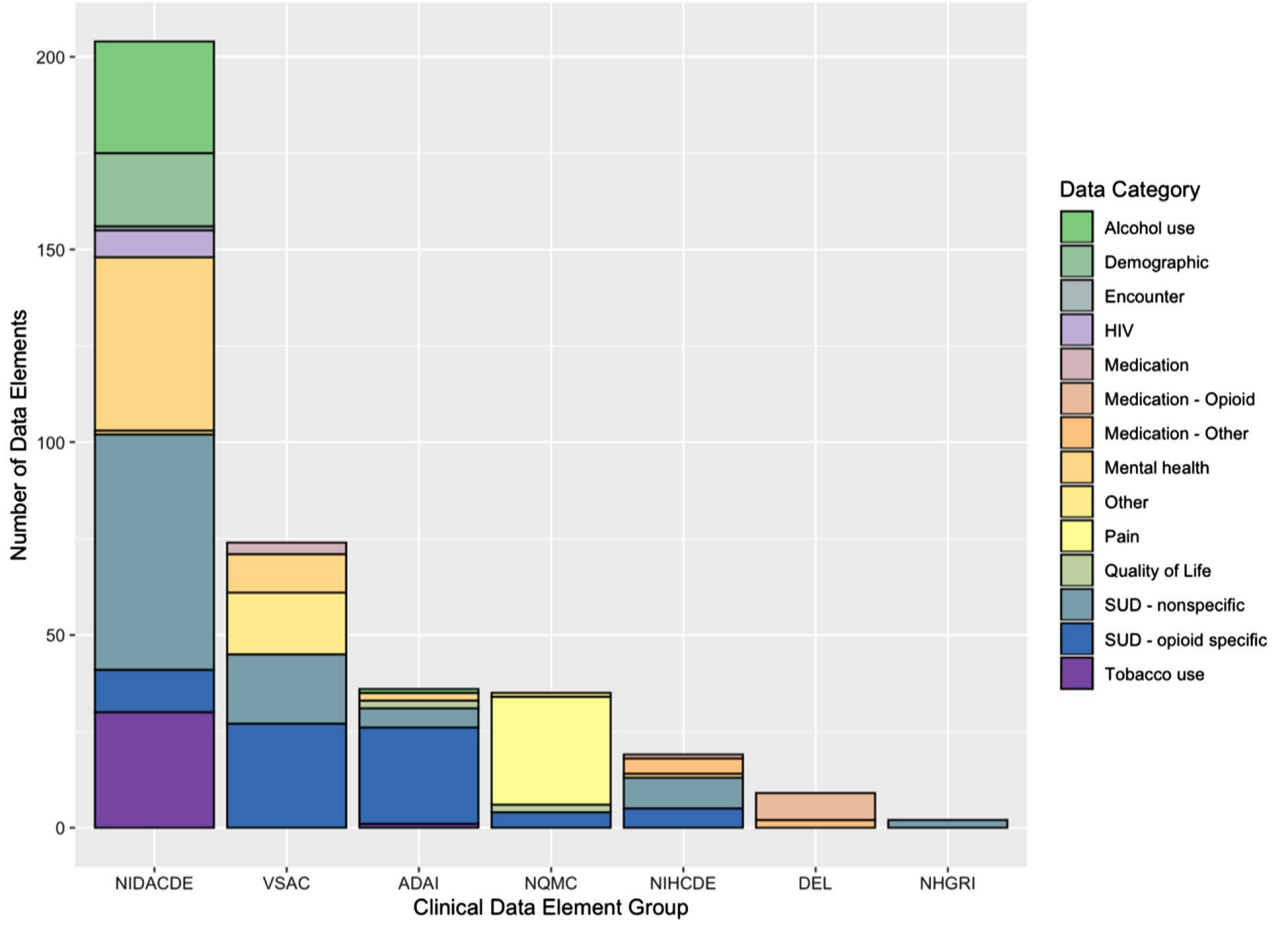
Distribution of clinical data element type by data system

Environmental scans of data dictionaries and databases on seven publicly available websites identified 379 CDEs, including 175 CDEs captured using the search term “opioid” and 204 contained within the NIDA CDEs. Based on manual review, only 84 (22%) of all CDEs identified were opioid-specific, while 93 (25%) were related to substance use disorder (SUD) and 202 (53%) were categorized as “other” (Table 1). The majority of opioid-specific CDEs were found in VSAC, which focused on intravenous drug use, pain medications and urine screening, and the Washington ADAI, which included clinical instruments such as the Clinical Opiate Withdrawal Scale and Opioid Craving Scale. When comparing 305 CDEs with related data elements contained within 9 common health data code systems in VSAC (e.g., CPT, ICD10, LOINC, etc.) for a combined total of 2745 potential matches, 61% had a partial match, 38% had no matches and less than 1% had an exact match with VSAC data code systems (Table 2). Overall, we found substantial variability in the types of clinical data elements available in each major data system with limited overlap (Fig. 2). Many CDE groups were dominated by one data category (e.g. NQMC) and few capture data elements from a wide variety of data categories well (e.g. NIDA CDEs). Notably, the NQMC included many CDEs specific to pain and quality of life but virtually none specific to mental health, which is captured by the NIDA CDEs, and no medications which are uniquely captured by the CMS DEL. No single data system includes CDEs across all major data element types such as SUD, OUD, medication and mental health. A comprehensive summary of categorized data elements is available in Additional file 1: Appendix S1.

**Table 1:** Opioid specificity of clinical data elements identified in environmental scan

Category	Data code systems							
	VSAC n (%)	CMS DEL n (%)	NQMC n (%)	NIH CDE n (%)	ADAI n (%)	PhenX n (%)	NIDA CDEs n (%)	Total n (%)
Opioid specific	27 (36)	9 (100)	1 (3)	12 (63)	24 (67)	0 (0)	11 (5)	84 (22)
SUD nonspecific	16 (22)	0 (0)	0 (0)	2 (11)	12 (33)	2 (100)	61 (30)	93 (25)
Other	31 (42)	0 (0)	34 (97)	5 (26)	0 (0)	0 (0)	132 (65)	202 (53)
Total	74 (100)	9 (100)	35 (100)	19 (100)	36 (100)	2 (100)	204 (100)	379 (100)

**Table 2:** Summary of VSAC matches by data code system

Data code system	Exact n (%)	Partial n (%)	None n (%)	Total n
NIDA CDE	9 (1)	1275 (69)	552 (30)	1836
CMS DEL	0 (0)	59 (73)	22 (27)	81
NQMC	0 (0)	0 (0)	315 (100)	315
NIH CDE	0 (0)	91 (53)	80 (47)	171
ADAI	0 (0)	252 (78)	72 (22)	324
PhenX	0 (0)	14 (78)	4 (22)	18
Total	9 (1)	1691 (61)	1045 (38)	2745

The literature review identified 38 articles for analysis (Additional file 2: Appendix S2), which described observational research, expert consensus/review publications and a limited set of experimental studies. The vast majority of studies were not directly reporting CDEs but rather included outcomes or cohort definitions that were descriptive of a CDE and suitable for consideration for future data infrastructure work.

Overall, the literature review identified a total of 237 CDEs that could potentially be OUD related, of which 225 (95%) were diagnosis-based and not opioid specific. These included descriptions of CDEs for other SUD such as alcohol use as well as diagnosis codes for concomitant mental health conditions. No standard or consistent diagnostic CDE definitions were used across the studies further indicating the lack of consensus or standard vocabularies for OUD CDEs.

## Discussion

This environmental scan and literature review revealed several notable gaps in the digital data infrastructure necessary for EHRs to support research on OUD. First, despite the increasing ubiquity of EHR data standards, we found substantial fragmentation in the design and construction of OUD-related CDEs. Value sets that are posted and curated within the NLM VSAC increasingly represent a centralized set or list of potential CDEs that define clinical concepts to support effective and interoperable health information exchange [19]. However, the value sets we identified are often limited to a single data type or data, which limits use across data systems and in turn exacerbates gaps in CDE capture of clinical concepts. For example, while diagnostic codes of OUD and medication-based value sets that could be used to identify OUD are independently present in the VSAC, the lack of data integration results in multiple OUD definitions of poor sensitivity and/or specificity. For OUD research initiatives to yield broadly generalizable results, future work must either develop validated cross walks between data sources (e.g. linking specific SNOMED concepts to ICD-10 diagnostic codes) or more likely, hybrid definitions that integrate multiple datatypes to characterize a clinical concept such as “opioid overdose” in a manner that leverages the strengths and accommodates the limitations of disparate electronic data systems and ontologies [20–22].

Second, we found little OUD-specific CDEs in existing data dictionaries and systems. Given high rates of co-occurrence, many substance use disorder CDEs are OUD-relevant **[**23], yet few CDEs effectively capture OUD-specific data needed for most research initiatives. For example, most NIDA CDEs relevant to OUD were initially developed or designed to assess SUD more broadly or for other substances such as alcohol [23]. In addition, while many medication CDEs exist related to opioids, few distinguished between opioid prescribing outside the hospital-based setting and within the hospital setting. Even fewer CDEs distinguish between the prescribing of opioids for episodic or acute conditions and chronic purposes. This is an important distinction for the development of future opioid related quality measures and research, as the gaps in current data infrastructure preclude many important observational or epidemiological analyses impossible without the opioid drug and OUD element specificity needed by investigators. Additionally, while we recognize that the number of opioid-specific CDEs is limited by the pool of data included in this review, when matching the NIDA CDEs—which specifically includes data relevant to substance use—there were still very few (n = 11) data elements specific to opioids.

Third, we found that traditional resource sources such as peer-reviewed publications contain few CDEs ready to use for existing data systems. Most research regarding structured data and patient-reported outcomes has utilized non-electronic data sources such as chart review or surveys, or low-fidelity sources such as insurance claims, and has also acknowledged notable limitations in data definitions due to the paucity of standard CDEs and definitions. Future data infrastructure efforts will need to rely on non-traditional data sources to identify CDEs and federal and state informatics initiatives to identify standards and be flexible to adapt non-electronic tools to electronic applications [24].

## Conclusions

Despite the increasing ubiquity of EHR data standards, we found substantial fragmentation in the design and construction of OUD related CDEs and little OUD specific CDEs in existing data dictionaries, systems and literature. Future work should leverage existing structured data elements to create standard workflow processes to improve OUD data capture in EHR systems.

## Supplementary information

**Additional file 1. **Summary of categorized data elements identified by environmental scan mapped to common data elements of Value Set Authority Center.

**Additional file 2. **Detailed results of literature review.

## Data Availability

All data generated or analyzed during this study are included in this published article and its Additional files 1, 2.

## Abbreviations

ADAI: Alcohol and Drug Abuse Institute
CDCREC: Centers for Disease Control and Prevention Race and Ethnicity Code Set
CDE: Common data element
CMS: Centers for Medicare and Medicaid
CPT: Current Procedural Terminology
DEL: Data element library
ED: Emergency department
EHR: Electronic health record
HCPCS: Healthcare Common Procedure Coding System
ICD10: International Classification of Diseases, 10th Revision
ICD9: International Classification of Diseases, 9th Revision
LOINC: Logical Observation Identifiers Names and Codes
NDFRT: National Drug File – Reference Terminology
NHGRI: National Human Genome Research Institute
NIDA: National Institute of Drug Abuse
NIH: National Institutes of Health
NQMC: National Quality Measures Clearinghouse
OUD: Opioid use disorder
SNOMED CT: Systematized Nomenclature of Medicine Clinical Terms
SUD: Substance use disorder
VSAC: Value Set Authority Center
